# Description of antibiotic use variability among US nursing homes using electronic health record data

**DOI:** 10.1017/ash.2021.207

**Published:** 2021-12-07

**Authors:** Sarah Kabbani, Stanley W. Wang, Laura L. Ditz, Katryna A. Gouin, Danielle Palms, Theresa A. Rowe, David Y. Hyun, Nancy W. Chi, Nimalie D. Stone, Lauri A. Hicks

**Affiliations:** 1 Centers for Disease Control and Prevention, Atlanta, Georgia, United States; 2 PointClickCare, Mississauga, Ontario, Canada; 3 Northwestern University Feinberg School of Medicine, Chicago, Illinois, United States; 4 The Pew Charitable Trusts, Washington, DC, United States

## Abstract

**Background:**

Antibiotics are frequently prescribed in nursing homes; national data describing facility-level antibiotic use are lacking. The objective of this analysis was to describe variability in antibiotic use in nursing homes across the United States using electronic health record orders.

**Methods:**

A retrospective cohort study of antibiotic orders for 309,884 residents in 1,664 US nursing homes in 2016 were included in the analysis. Antibiotic use rates were calculated as antibiotic days of therapy (DOT) per 1,000 resident days and were compared by type of stay (short stay ≤100 days vs long stay >100 days). Prescribing indications and the duration of nursing home-initiated antibiotic orders were described. Facility-level correlations of antibiotic use, adjusting for resident health and facility characteristics, were assessed using multivariate linear regression models.

**Results:**

In 2016, 54% of residents received at least 1 systemic antibiotic. The overall rate of antibiotic use was 88 DOT per 1,000 resident days. The 3 most common antibiotic classes prescribed were fluoroquinolones (18%), cephalosporins (18%), and urinary anti-infectives (9%). Antibiotics were most frequently prescribed for urinary tract infections, and the median duration of an antibiotic course was 7 days (interquartile range, 5–10). Higher facility antibiotic use rates correlated positively with higher proportions of short-stay residents, for-profit ownership, residents with low cognitive performance, and having at least 1 resident on a ventilator. Available facility-level characteristics only predicted a small proportion of variability observed (Model R^2^ version 0.24 software).

**Conclusions:**

Using electronic health record orders, variability was found among US nursing-home antibiotic prescribing practices, highlighting potential opportunities for targeted improvement of prescribing practices.

Antibiotics are frequently prescribed inappropriately in nursing homes,^1,2^ and they pose a risk of adverse drug events^3^and increased risk of infection with multidrug-resistant organisms.^4,5^ The Centers for Disease Control and Prevention’s (CDC) Core Elements of Antibiotic Stewardship highlight the importance of tracking antibiotic use to optimize prescribing practices.^6,7^ The Centers for Medicare & Medicaid Services (CMS) revised the Requirements for Participation in Medicare for Long-Term Care in 2016, requiring antibiotic stewardship programs that include a system to monitor antibiotic use.^8^ However most nursing homes lack the infrastructure and expertise to effectively analyze antibiotic use data.^9^ Unlike acute-care hospitals,^10^ few studies describe antibiotic use in this setting, and benchmarks to evaluate antibiotic use across nursing homes are not available. Electronic health records (EHR) are increasingly available in US nursing homes and are used for medication orders,^11–13^ and they may be a useful data source to track and report antibiotic use in nursing homes.^9^ The objectives of this analysis were to use EHR antibiotic orders to describe national and facility-level antibiotic use rates and assess facility-level variability in a large cohort of US nursing homes.

## Methods

PointClickCare is a health technology company that provides cloud-based software to >21,000 long-term and acute/post-acute care providers and covered 8,200 providers in 2016. A retrospective observational cohort study design was used to describe antibiotic use using EHR orders in 1,664 nursing homes that previously consented to de-identification and aggregation of their data from January 1 to December 31, 2016. Resident demographics were reported.

### Description of antibiotic use

The proportion of unique residents receiving antibiotics was reported. One antibiotic day of therapy (DOT) represents the order of a single antibiotic on a given day. Systemic routes of administration were included (oral, intravenous, and intramuscular). Antibiotic DOT were classified by class (Table S1). If the end date for the antibiotic order was missing, the discontinuation date was used, if both were missing, the order was excluded (0.8% of orders). The number of resident days was determined by census data, and antibiotic rates were calculated as DOT per 1,000 resident days. Nursing home stays were defined as continuous if the gap between discharge and readmission was <3 days. Nursing home stays were classified into short stays (≤100 days duration) and long stays (>100 days duration). Antibiotic use rates were reported by agent, class, route, type of nursing home stay, and time from nursing home admission to the first antibiotic order. An antibiotic course combined orders for the same drug with a prescription gap of ≤1 day. Duration of antibiotic courses were reported. Nursing home–initiated courses were defined as courses ordered on or after day 3 of admission.

Indications were transcribed into the EHR by the prescriber when an antibiotic was ordered; 80% of the indications were entered as free text and 20% were entered using *International Classification of Disease, Tenth Revision* (ICD-10) diagnoses. We were unable to directly determine which indications were prescribed for prophylaxis or treatment; antibiotic courses ≤1 day and >42 days in duration were considered prophylaxis and were excluded from classification under specific infection type. Data mining of all remaining indications was conducted to classify 96.7% of antibiotic courses into 5 infection categories: genitourinary; respiratory; skin, soft-tissue, and musculoskeletal; gastrointestinal or intraabdominal; and other. For example, terms such as “UTI,” “URIN,” “bladder,” “kidney,” “renal,” “prostate,” and “pyelo” in the indication field were grouped under genitourinary infections. Dental prescribing was classified under skin, soft-tissue, and musculoskeletal infections. If >1 indication was identified, the antibiotic course was classified into the infection category other than genitourinary infection. Prescribing practices by infection type were described by drug, number of courses, median, and interquartile range (IQR) of course durations initiated in nursing homes, excluding prophylaxis.

### Facility-level antibiotic use rates

A univariate and multivariate linear regression analysis was performed to evaluate the variation in facility-level antibiotic use rates. Medians and IQRs of facility-level antibiotic use rates and proportion of short-stay residents are reported. The main exposure variable was proportion of short-stay residents and was categorized at the median (75% or higher). Facility-level correlates of resident health and facility characteristics publicly available through LTCfocus^14^ and CMS Nursing Home Compare^15^ were identified and described for all nursing homes with available data (Supplementary Table S2). Facility-level characteristics of nursing homes included in this analysis were compared to all national nursing homes available in the data sets. Continuous covariates were categorized at the median. Comparison between individual covariates and facility-level antibiotic use was performed using the χ^2^ test or the Fisher exact test where appropriate for categorical variables and the Wilcoxon-Mann-Whitney test for continuous variables. Reported *P* values with α (significance level) <.05 were included in the multivariate linear regression model for facility-level antibiotic use rates as the outcome and the proportion of short-stay residents as the main exposure.^4^ Interaction was assessed using the Breslow-Day method. Because “facility direct care hours per resident day” was found to have an association with type of nursing home stay, it was included as an interaction term in the model for antibiotic use rates. Collinearity of covariates was also assessed. A final model was built using stepwise selection methods. Model R^2^ was reported to assess the proportion of variance of facility-level antibiotic use explained by the variables included in the model. Statistical analyses were performed using SAS version 9.4 software (SAS Institute, Cary, NC).

### Study cohort

PointClickCare utilized data from clients that had previously consented (in their Business Associated Agreement, BAA) to both deidentification and aggregation of their data, without any restrictions. Under the terms of the BAAs, PointClickCare complied with obligations under Health Insurance Portability and Accountability (HIPPA) and the Health Information Technology for Economic and Clinical Health (HITECH) Act regarding the use and disclosure of protected health information. No protected health information was shared with the CDC, and the CDC did not have access to resident-level data. Summarized aggregated antibiotic use data were shared with CDC collaborators. The National Center for Emerging and Zoonotic Infectious Diseases reviewed the protocol and determined that the project did not meet the definition of research and was exempt from human subject review.

## Results

Resident demographics and antibiotic use data for the 1,664 study nursing homes are listed in Table 1. Among the cohort of 309,884 US nursing-home residents, 167,647 (54%) received an antibiotic, and the antibiotic use rate overall was 88 DOT per 1,000 resident days. During 2016, a total of 254,063 residents were considered short-stay and contributed to ∼10 million resident days, whereas the 55,821 long-stay residents contributed to 31 million resident days. Short- and long-stay residents had similar demographic characteristics, but higher antibiotic use rates were observed in short-stay residents (330 DOT per 1,000 resident days) compared to long-stay residents (30 DOT per 1,000 resident days). A larger proportion of long-stay residents received antibiotics over the full year (79% vs 49%) (Table 1).

**Table 1.** Resident Demographics and Characteristics of Antibiotic Use in 1,664 Nursing Homes in 2016

Variable	Total		Short-Stay Nursing Home Stay ≤100 Days		Long-Stay Nursing Home Stay >100 Days	
	(Residents = 309,884) (ResidentDays = 41,589,528)		(Residents = 254,063) (Resident Days = 10,227,757)		(Residents = 55,821) (Resident Days = 31,361,781)	
	No. or Median	%^a^	No.^a^	%^a^	No.^a^	%^a^
**Resident demographic characteristics**						
Age, median y and IQR	77	67–86	77	67-85	77	68-87
Sex, female	179,550	58	145,854	57	33,696	60
**Antibiotic use**						
Unique residents receiving an antibiotic	167,647	54	123,805	49	43,842	79
Total antibiotic DOT	504,268	NA	256,338	NA	247,930	NA
Antibiotic DOT per 1,000 resident days	88	NA	330	NA	30	NA
**Antibiotic days of therapy by route**						
Oral	408,161	81	195,166	76	212,995	86
Intravenous	74,876	15	54,408	21	20,468	8
Intramuscular	21,231	4	6,764	3	14,467	6
**Antibiotic days of therapy by class**						
Fluoroquinolones	1,041,883	18	537,642	18	504,241	18
Cephalosporins	1,023,666	18	544,644	18	479,022	18
Other	636,889	11	426,163	14	210,726	8
Urinary anti-infectives	524,887	9	177,240	6	347,647	12
Sulfa drugs	512,297	9	217,955	7	294,342	10
Extended activity β lactams	487,812	8	290,731	10	197,081	7
Tetracyclines	477,305	8	227,648	8	249,657	9
Glycopeptides	384,872	7	288,788	10	96,084	3
Penicillins	327,895	6	121,369	4	206,526	7
Macrolides	245,842	4	101,577	3	144,265	5
Lincosamides	141,584	2	63,203	2	78,381	3
**Antibiotic days of therapy by drug (top 5)**						
Levofloxacin	556,469	10	295,473	10	260,996	9
Trimethoprim-sulfamethoxazole	511,230	9	217,188	7	294,042	10
Ciprofloxacin	464,508	8	231,080	8	233,428	8
Cephalexin	449,720	8	200,030	7	249,690	9
Doxycycline	399,136	7	189,967	6	209,169	7

Note. IQR, interquartile range; DOT, days of therapy; NA, not available.

a Unless otherwise specified as median and IQR.

During 2016, 49% of short- and long-stay nursing home residents receiving an antibiotic were prescribed 1 antibiotic course, 35% received 2–3 courses and 16% received ≥4 courses. Most antibiotics were administered via oral route (81%), 15% were intravenous, and 4% were intramuscular. Fluoroquinolones, cephalosporins, and urinary anti-infectives were the most commonly prescribed antibiotic classes; levofloxacin, trimethoprim-sulfamethoxazole, and ciprofloxacin were the most commonly prescribed agents (Table 1). Of all antibiotic courses prescribed in the nursing home, 29% were started on or within 2 days of admission. The duration of over half of nursing home–initiated antibiotic (started on or after the day 3 of admission) courses was ≤7 days (Supplementary Table S2). However, 14.4% of all nursing home–initiated courses were classified as prophylaxis (duration ≤1 day and >42 days) and contributed to 23.3% of total DOTs (Supplementary Table S2).

Among nursing home–initiated antibiotic courses, genitourinary infections were the most common infection category and accounted for 33.5% of courses. Notably, 61.0% of antibiotic courses prescribed for gastrointestinal and intra-abdominal infections were for *Clostridioides difficile* infection. Median duration of most nursing home-initiated courses was 6–8 days (Table 2).

**Table 2.** Duration of Nursing-Home Initiated^a^ Antibiotic Courses^b^ for Top Five Antibiotics by Infection Type^c^ in 1,664 Nursing Homes in 2016

Antibiotic	Total Courses		Course Duration (Days)	
	No.	%	Median	IQR
Total	436,619	NA	7	5–10
**Genitourinary infections**				
Total	146,239	NA	7	5–8
Ciprofloxacin	32,042	22	7	4–8
Nitrofurantoin	22,995	16	8	6–9
Trimethoprim-sulfamethoxazole	18,492	13	7	5–8
Levofloxacin	12,736	9	6	4–7
Cephalexin	12,351	8	7	5–8
**Respiratory infections**				
Total	100,165	NA	7	4–8
Levofloxacin	32,966	33	7	5–8
Azithromycin	17,879	17	5	4–5
Amoxicillin-Clavulanic Acid	9,768	10	8	5–10
Doxycycline	7,359	7	8	6–10
Ceftriaxone	4,472	4	5	3–7
**Skin, soft-tissue, and musculoskeletal infections**				
Total	81,488	NA	8	6–11
Cephalexin	17,476	21	8	6–11
Doxycycline	9,918	12	9	7–11
Trimethoprim-sulfamethoxazole	9,186	11	8	7–11
Vancomycin (IV)	6,005	7	7	4–13
Clindamycin	5,960	7	8	6–11
**Gastrointestinal and intraabdominal infections**				
Total	26,105	NA	9	6–13
Metronidazole	10,539	40	9	6–12
Vancomycin (oral)	8,428	32	10	6–14
Rifaximin	1,612	6	13	7–22
Ciprofloxacin	1,178	6	7	7–10
Levofloxacin	683	3	7	5–8


Note. IQR, interquartile range; IV, intravenous.

a Nursing home-initiated courses, first antibiotic order start date ≥3 days after nursing home admission.

b Antibiotic course, all orders for the same drug with ≤ 1 day gap.

c Prophylaxis, defined as courses ≤ 1 day or >42 days, excluded.

The median facility-level antibiotic use rate was 81 DOT per 1,000 resident days (IQR, 43–140). The median proportion of short-stay residents at a facility was 75% (IQR, 62%–86%). The median antibiotic prescribing rate in facilities with a proportion of short-stay residents ≥75% was 90 DOT per 1,000 resident days compared to 38 DOT per 1,000 resident days in facilities where the proportion of short-stay residents was <75%. Aggregated resident- and facility-level covariates significantly associated with facility-level antibiotic use are shown in Table S3. In a multivariate linear regression model, higher antibiotic use rate correlated positively with the following facility-level characteristics: proportion of short-stay residents ≥75%, for-profit ownership, higher proportion of residents with low cognitive performance scale ≥50%, facility proportion of long-stay residents with pressure ulcers ≥5% and having at least 1 resident on a ventilator. The model R^2^ was 0.24 (Table 3).

**Table 3.** Facility and Resident Characteristics Associated with Facility-Level Antibiotic Use in 1,664 Nursing Homes in 2016

Variables	Univariate Analysis		Multivariate Regression (Model R^2^ = 0.24)		
	Coefficient	*P* Value	Coefficient	*P* Value	Partial R^2^
Facility proportion of short-stay residents ≥75%	72.8	<.0001	42.2	<.0001*	0.117
**Facility location**					
Rural^1^	52.7				
Large rural	16.4	.023	−4.9	.386	0.004
Suburban	28.0	.003	−3.7	.613	0.005
Urban	49.1	<.0001	7.6	.117	0.044
Facility for profit ownership	22.5	<.0001	10.0	.0121*	0.003
**Facility no. of beds**					
<50	7.8	.236	7.5	.188	0.000
50–99^a^	76.3				
100–199	21.0	<.0001	5.9	.110	0.001
>199	16.9	.043	6.4	.370	0.001
Facility part of multifacility organization	8.7	.035	6.6	.056	0.001
Facility average resident age < 80 y	12.7	.001	1.7	.644	0.000
Facility nursing case mix index ≥1.3	21.3	<.0001	−1.8	.646	0.003
Facility with average resident activity of daily living score ≥16	13.2	.002	−6.1	.131	0.001
Facility proportion of residents with low cognitive performance scale ≥50%	26.2	<.0001	8.9	.010*	0.028
Facility with at least 1 resident on a ventilator	73.8	<.0001	53.8	<.0001*	0.009
Facility proportion of long-stay residents with urinary catheters ≥ 2.5%	8.0	.035	3.9	.217	0.001
Facility proportion of long-stay residents with pressure ulcers ≥ 5%	17.7	<.0001	9.9	.002*	0.003
Facility registered nurse staffing hours per resident day ≥0.43	24.8	<.0001	3.3	.327	0.008
Facility direct care hours per resident day	37.9	<.0001	1.9	.702	0.011
Interaction term with short stay			20.7	.002*	0.005

*P value <.05.

a Reference.

## Discussion

Antibiotic orders captured in EHR were used to describe antibiotic use in nursing homes at the facility and national levels. Median facility-level antibiotic use rates varied considerably and were only partially explained by facility and facility-level resident characteristics, highlighting potential opportunities for targeted improvement of prescribing practices.

More than half of all nursing-home residents were prescribed an antibiotic, as reported previously.^5,16^ Although antibiotic use rates were higher in short-stay residents,^4^ a larger cumulative proportion of long-stay residents receive antibiotics over time. Almost one-third of antibiotic prescriptions were prescribed on admission, highlighting the opportunities for discharge stewardship. A recent analysis reported that 23% of patients discharged from hospital to long-term care facilities were discharged on antibiotics, and antibiotic use was significantly associated with a 30-day emergency department visit and *C. difficile* infection within 60 days.^17^

Urinary and respiratory tract infections are the most common indications for antibiotic use in nursing homes, as shown previously,^18^ and fluoroquinolones are commonly prescribed.^5,19^ A high frequency of asymptomatic bacteriuria in combination with inappropriate culturing practices in nursing homes has been associated with antibiotic overprescribing.^20,21^ Overuse of antibiotics has been reported for nursing home residents with respiratory symptoms.^22,23^ Increasing awareness of adverse events associated with antibiotic use, review of medications in nursing home emergency kits, and implementation of antibiotic prescribing protocols and decision support tools can help optimize the treatment of infections in nursing home settings.^16,24^ After excluding courses classified as prophylaxis, the duration of almost half (48%) of all nursing home-initiated courses exceeded 7 days, similar to prescribing in nursing homes in Ontario, Canada, where 45% of prescriptions exceeded 7 days.^25^ With a reported 0.4% increase in the risk of antibiotic-related harm with every additional day increase of antibiotic use,^26^ and increasing evidence for the effectiveness of shorter antibiotic courses,^27^ optimizing treatment duration is critical for resident safety. Implementing standardizing antibiotic reviews when the clinical picture is clearer and more diagnostic information is available can optimize the selection and duration of antibiotic treatment and should be further adapted to nursing home workflows.^6,7^ Further evaluation of the effect of individual prescribing practices and the number of unique prescribers in a facility on the frequency of antibiotic prescribing is needed.^19,25,28^ Behaviorally targeted assessment and feedback interventions should be evaluated in nursing home settings.^29^

Even after adjusting for facility-level characteristics, the proportion of short-stay residents was found to be associated with higher facility-level antibiotic use rates, as shown previously.^4^ Median antibiotic use ate in facilities where cumulative proportion of short-stay residents ≥75% was almost 2.5 times that of facilities with a lower proportion of short-stay residents. These residents are at increased risk for infections following hospital discharge and should be adjusted for when developing facility-level benchmarks. Another notable finding is that having at least 1 ventilator bed in the facility was a strong predictor of antibiotic use. Ventilator-capable skilled-nursing facilities have been associated with increased risk of resident colonization of multidrug-resistant organisms underscoring the importance of infection control and antibiotic stewardship.^30^ Different measures of functional status have been evaluated in relation to development of infection and possible antibiotic use.^31^ In this analysis, higher measures of nursing care requirements were associated with higher rates of antibiotic use on univariate but not multivariate analysis. The case-mix index, which is used to determine reimbursement for nursing home care, has been associated with antibiotic use,^32^ and its role may have been minimized by the high proportion of short-stay residents in this analysis. Overall, facility-level characteristics only explained 24% of antibiotic use variability highlighting the need for additional analyses to further explore different variables and opportunities for improving antibiotic use.

This study had several limitations. Although some resident characteristics were captured at the facility level, these variables were not available at the resident-level and further evaluation of the influence of specific resident characteristics is needed. Also, prescriber-level data were not available and could not be assessed. A general description of indications was based on provider documentation and no validation was done. Misclassification of infections may limit the use of these data at a facility level without additional provider training and validation. Including general infection categories as part of EHR orders rather than free text may help identify opportunities for improvement and provide feedback for providers on their prescribing practices. Emergency room transfers or hospital admissions that were <3 days were not captured in this analysis; thus, the proportion of antibiotic courses that were initiated outside the nursing home may have been underestimated. Antibiotic orders, which may not always reflect actual administration, were described in this analysis. However, antibiotic orders may be a good proxy for prescribing behavior, especially for audit-and-feedback interventions. Although this analysis reflects data from 2016 and some prescribing practices may have changed over time, the analytic methods used in the analysis of EHR orders and identifying facility-level characteristics remain valuable and can serve as a baseline for assessing the effect of stewardship regulatory requirements. Although this analysis provides a description of a large subset of nursing homes, it may not be representative of national practices.

Tracking antibiotic prescribing in nursing homes is critical to assess opportunities for improvement, guide practice change, evaluate the impact of stewardship interventions and improve resident outcomes.^6,9^ Further evaluation and validation of EHR antibiotic use data and measures can support nursing homes in using these data in tracking and reporting antibiotic use. Other important potential next steps include evaluating data elements for risk adjustment of facility-level benchmarks,^31^ streamlining the generation of antibiotic use reports for individual facilities^33^ and assessing the impact of prescriber audit and feedback in this setting.^29^

## Supplementary material

For supplementary material accompanying this paper visit https://doi.org/10.1017/ash.2021.207. click here to view supplementary material
