# Supplementary material for: Description of antibiotic use variability among US nursing homes using electronic health record data
Source: Antimicrob Steward Healthc Epidemiol. 2021 Dec 7;1(1):e58. doi: 10.1017/ash.2021.207 (PMC9495428; doi:10.1017/ash.2021.207)
Supplement: Supplementary file 1 [file ashsup.zip › S2732494X21002072sup001.docx]

| **Table S2: Antibiotic Course Treatment Duration in 1,664 Nursing Homes in 2016** | | | | | | | | |
| --- | --- | --- | --- | --- | --- | --- | --- | --- |
|  | **All Antibiotic Courses** | | | | **Nursing-Home Initiated Courses^1^** | | | |
| **Course Duration** | **#** | **%** | **Days of Therapy** | **%** | **#** | **%** | **Days of Therapy** | **%** |
| **1 day** | 60,414 | 12.1% | 60,414 | 1.8% | 45,885 | 12.8% | 45,885 | 1.5% |
| **2-7 days** | 231,258 | 46.4% | 1,123,051 | 33.0% | 159,992 | 44.7% | 784,935 | 25.3% |
| **8-14 days** | 167,269 | 23.5% | 1,617,470 | 47.6% | 129,432 | 36.2% | 1,242,625 | 40.1% |
| **15-42 days** | 30,722 | 6.2% | 670,508 | 19.7% | 16,967 | 4.7% | 347,168 | 11.2% |
| **>42 days** | 9,081 | 1.8% | 1,110,304 | 32.7% | 5,451 | 1.5% | 677,149 | 21.9% |
| 1. Nursing home-initiated courses, first antibiotic order start date ≥3 days after nursing home admission | | | | | | | | |

| **Table S3: Data source, definition, and classification of 1,664 nursing home facility characteristics and comparison to national facility characteristics, 2016** | | | | | | |
| --- | --- | --- | --- | --- | --- | --- |
| **Variable^1^** | **Definition and Classification** | **Source** | **Study Facilities**  **N=1,664** | | **National Facilities**  **N= 15,164** | |
|  |  |  | N/Median | IQR^2^** | N/Median | IQR^2^ |
| **Facility Antibiotic Use Rate** | Days-of-Therapy/1,000 Resident-Days  (outcome) | PCC | 81 | 43-140 | - | - |
| **Facility Proportion of Short-Stay Residents** | Nursing Home Stay ≤ 100 days  (exposure) | PCC | 74.88% | 61.54-85.71% | - | - |
| **Facility Location** | Rural Urban Commuting Area Codes (RUCA) | LTCFocus^3^ |  |  |  |  |
| **Rural** | RUCA=1, 1.1 (reference) |  | 20.0% | - | 18.2% | - |
| **Large Rural** | RUCA=2, 2.1, 3 |  | 13.2% | - | 14.2% | - |
| **Suburban** | RUCA=4, 4.1, 4.2, 5, 5.1, 5.2, 6, 6.1 |  | 6.0% | - | 7.4% | - |
| **Urban** | RUCA=7, 7.1-7.4, 8-8.4, 9, 9.1, 10-10.6 |  | 59.6% | - | 59.9% | - |
| **Facility Type of Ownership** | For-Profit vs. Other | LTCFocus | 72.1% | - | 69.7% | - |
| **Facility Number of Beds** |  | LTCFocus |  |  |  |  |
| **<50** |  |  | 4.6% | - | 0.2% | - |
| **50-99** | (reference) |  | 11.2% | - | 12.0% | - |
| **100-199** |  |  | 37.4% | - | 37.6% | - |
| **>199** |  |  | 40.7% | - | 44.1% | - |
| **Facility Organizational Structure** | Multi-facility vs. Independent | LTCFocus | 60.1% | - | 57.9% | - |
| **Facility Average Resident Age** | < 80 vs. ≥ 80 years | LTCFocus | 80.32 | 76.30-83.83 | 80.70 | 76.29-84.40 |
| **Facility Nursing Case Mix Index** | ≥ 1.3 vs. <1.3 | LTCFocus | 1.28 | 1.22-1.36 | 1.29 | 1.21-1.37 |
| **Facility Average Activity of Daily Living Score** | ≥ 16 vs. <16 | LTCFocus | 16.86 | 15.62-17.97 | 17.08 | 15.70-18.25 |
| **Facility Proportion of Residents with Low Cognitive Performance Scale** | ≥ 50% vs. <50% | LTCFocus | 54.0% | 45.76-62.22% | 54.0% | 44.27-62.62% |
| **Facility with at least one resident on a ventilator** | Yes vs. No | LTCFocus | 1.5% | - | 1.6% | - |
| **Facility Proportion of Long-stay Residents with Urinary Catheters** | 2.5% vs <2.5% | CMS^4^ | 2.36% | 1.14-3.94% | 2.33% | 1.15-3.84% |
| **Facility Proportion of Long-stay Residents with Pressure Ulcers** | ≥ 5% vs. < 5% | CMS | 5.10% | 3.04-7.71% | 5.16% | 3.07-7.69% |
| **Facility Registered Nurse Staffing Hours per Resident Day** | ≥ 0.43 vs. < 0.43 | LTCFocus | 0.429 | 0.273-0.603 | 0.402 | 0.242-0.623 |
| **Facility Direct Care Hours per Resident Day** | ≥ 3.5 vs. <3.5 | LTCFocus | 3.433 | 2.991-3.970 | 3.536 | 3.077-4.084 |
| 1. Other variables considered include: CMS Regions, Whether a facility was Hospital-Based, Facility RN Average Staffing Hours per Patient Day, Facilty Proportion of Residents with Mid Cognitive Performance Scale, Facilty Proportion of Residents with High Cognitive Performance Scale, Facility Observed 30-Day Rehospitalization Rate, Facility Overall Five-Star Rating, Facility Survey Five-Star Rating, Facility Quality Five-Star Rating, Facility Staffing Five-Star Rating, Facility RN Staffing Five-Star Rating, Facility Average Census 2. IQR Interquartile Range 3. Shaping Long Term Care in America Project at Brown University funded in part by the National Institute on Aging (1P01AG027296) <http://www.ltcfocus.org/> 4. Centers for Medicare & Medicaid Services. *Nursing Home Compare* <https://www.medicare.gov/nursinghomecompare/search.html> | | | | | | |
